# Deaf-Mutes

**Published:** 1896-08

**Authors:** 


### DEAF-MUTES.

It is not so well recognized as it ought to be that a large proportion of deaf mutes retain a certain amount of hearing power. We say retain, because the majority of these unfortunates are sufferers from the results of aural disease developed before speech has been thoroughly acquired. It is true that in children of deficient intellect speech may be practically impossible, and that these cases are sometimes hard to separate from true deaf-mutism.

In a minority of cases the defect is congenital, and it seems to run in families, without, however, being commonly hereditary. Consanguinity in parents has a more decided influence, and syphilis, maternal impressions, alcoholism, bad hygiene, etc., are probably important factors in its production.

In this country and on the continent of Europe intracranial disease is probably the most common cause of acquired deaf-mutism, and cerebro-spinal meningitis is specially prominent in statistics. But disease of the external, middle, or inner ear, producing deafness, will also produce mutism when the patient is insufficiently advanced in intellect on account of age or natural deficiency. A child who loses his hearing, or becomes distinctly deaf, before the age of four will almost certainly also lose the power of speech, and between four and seven the progress as regards speech will mainly depend on the child's intellectual activity. Totally deaf mutes are the exception, though authors differ as to the exact percentage. The practical point is whether or not sensibility for sound exists to such a degree as can be utilized in teaching. Thus Love found that such sensibility existed in 25.27 of his cases, and Walker (*Med. Fortnightly,* March 2, 1896, and *Annals of Ophthal. Otology,* etc. May, 1896, p. 515,) found that 11 per cent. of the children whom he examined had 30 per cent. and over of hearing. If children could make as much use of their defective ears as adults can these would all have comparatively little trouble, for adults can get along with 7 per cent. of hearing power with artificial aid, but children are unable to put forth the necessary exertion. In New York 5 per cent. are taught through the ear, and in Nebraska an effort is being made so to teach 34 per cent.

When speech cannot be taught through the ears, one must fall back, when the child is above seven years old, upon the system of oral instruction and lip-reading, which consists in teaching the pupil to speak by imitation of the movements of the lips and tongue, modulation being secured by allowing him to feel with his fingers the vibrations of the teacher's larynx. For hearing is substituted observation of the speaker's lips.

According to Hartmann, a few pupils learn so successfully that a stranger does not notice any defect; one-third, though their speech is peculiar, can yet converse with any one; a second third can be understood by acquaintances alone, while the rest turn again to the language of signs.

